# Hand‐held optical coherence tomography imaging in children with anterior segment dysgenesis

**DOI:** 10.1111/aos.13053

**Published:** 2016-04-30

**Authors:** Anastasia V. Pilat, Viral Sheth, Ravi Purohit, Frank A. Proudlock, Samira Anwar, Irene Gottlob

**Affiliations:** ^1^ Department of Neuroscience, Psychology and Behaviour The University of Leicester Ulverscroft Eye Unit Leicester UK; ^2^ Ophthalmology Group University of Leicester Leicester UK

**Figure 1:**
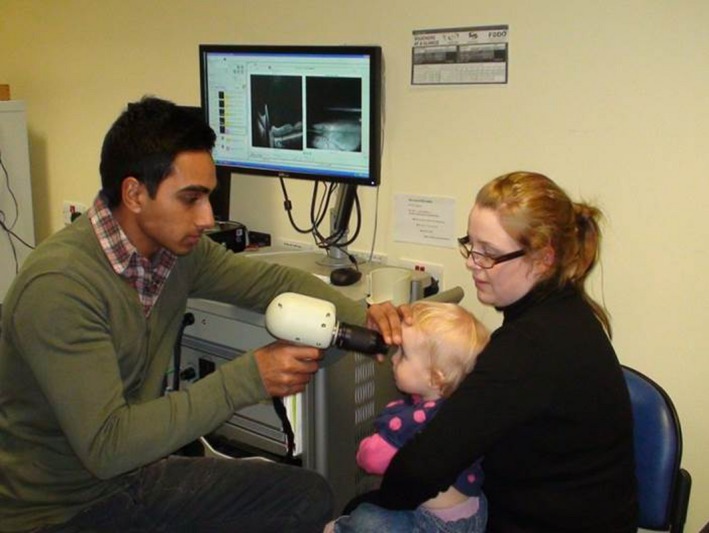
Optical coherence tomography (OCT) was performed in all participants without anaesthesia or sedation. A hand‐held spectral domain portable device (Bioptigen Inc., Research Triangle Park, NC), 840 nm wavelength and 2.6 mm theoretic axial resolution, was used. The advantage of the device is a separate probe that can be widely used in paediatric practice as patients’ fixation is not required.

In this study, we investigated the potential of hand‐held optical coherence tomography (HH‐OCT; Fig. 1) to improve diagnosis in anterior segments (AS) by visualizing the anterior and posterior eye structures without general anaesthetic (GA) or sedation.

Patients were prospectively recruited from paediatric clinics at the Leicester Royal Infirmary (Table 1).

**Table 1:** Age, gender, ethnicity and clinical data of the patients with congenital anterior segment abnormalities on the date of optical coherence tomography examination

Patient's number	Pathology	Age (years)	Gender	Ethnicity	LogMar visual acuity		Refraction (spherical equivalent)		Intraocular pressure		Associated ophthalmic pathology	Associated general pathology	Clinical findings	OCT findings
					RE	LE	RE	LE	RE	LE				
1	Axenfeld's anomaly	5	F	C	**0.1**	**0.1**	+1.0	+1.0	13	14	–	–	Irregular circular white structure underneath of the endothelium	Prominent anteriorly displaced Schwalbe line
2	ICE syndrome	5	F	C	**0.3**	**0.9**	−1.0	−0.5	14	14	Left esotropia, MLN	Arthrogryposis multiplex Congenital Hypothyroidism	Bilateral paracentral corneal opacities; round white structure underneath the endothelium	Smooth epithelial contour and uneven corneal endothelium; a ‘ring’ located 1–2 mm from the trabecular meshwork in BE
3	Peter's anomaly	6	M	C	0.4	**POL**	+1.25	–	13	12	Left esotropia, MLN	–	Iridocorneal and corneolenticular adhesions associated with corneal/lens opacity	Irregular corneal structure with pericentral corneal thickening on the affected side with iridocorneal adhesion; no iris folds on the LE; RE cup diameter/depth smaller than in control
4	*PAX6* mutation with aniridia	14	M	C	**0.5**	**0.4**	+2.25	+2.00	15	15	Infantile nystagmus	–	Aniridia and small central lens opacities bilaterally	Missing central part of the iris with hypoplastic stump 360°; foveal hypoplasia with continuation of RNFL, ganglion cell, inner nuclear and inner plexiform layers
5	Iris, optic nerve coloboma	12	M	C	**0.85**	0.2	−1.0	–	14	14	–	–	LE iris and choroidal coloboma	Hypoplastic irides with a remaining stump; large ON with RNFL thinning; large LE foveal pit
6	Primary childhood glaucoma	4	M	C	**0.9**	0.1	−10.0	+1.0	9	12	–	–	Large RE corneal diameter with haze and Haab's striae; abnormal iris insertion on gonio; cup/disc ratio of 0.6	Additional tissue on the endothelium RE; abnormal iris insertion; increased cup/disc ratio with RNFL thinning

BE = both eyes, F = female, ICE = iridocorneal endothelial syndrome, LE = left eye, M = male, MLN = manifest latent nystagmus, OCT = opticalcoherence tomography, ON = optic nerve, POL = perception of light, RE = right eye, RNFL = retinal nerve fibre layer.

Visual acuity in the affected eye is shown in bold.

**Figure 2:**
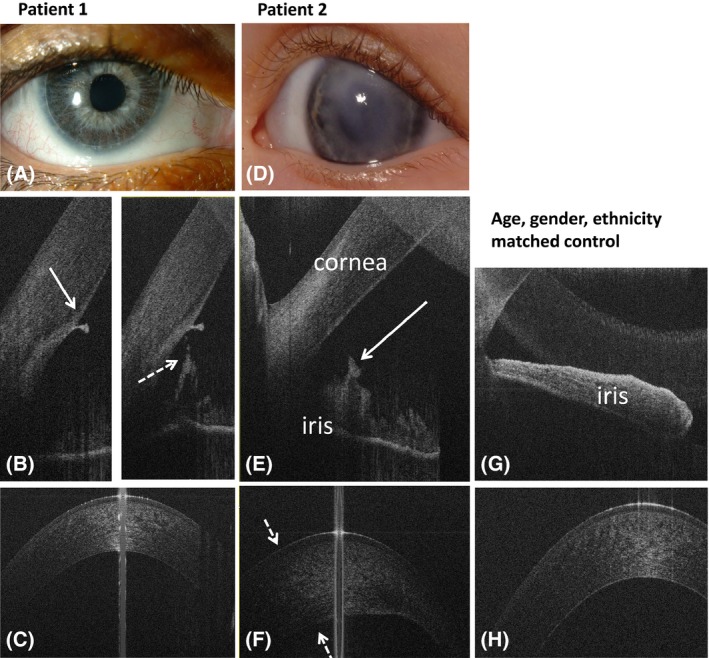
Anterior segment photography (A, D) and spectral domain optical coherence tomography (SDOCT) of anterior tomograms of case 1 with Axenfeld's anomaly (A–C) and case 2 with iridocorneal endothelial (ICE) syndrome (D–F) and a healthy control subject (G, H). Case 1 has an irregular circular white structure located underneath of the endothelium anteriorly to the limbus seen on slit‐lamp photography (A). The white arrow on figure B (right) shows an anteriorly displaced Schwalbe line. The dotted arrow on figure B (left) indicates zone of an iridocorneal adhesion on the same patient. Figure C shows normal corneal structure on OCT, similar to the cornea of the control subject (H). Patient with ICE syndrome (D–F) had an iris ‘band’ located in 1–2 mm from the trabecula (arrow on figure E) and an irregular structure of the cornea. Dotted arrows on figure F show the thickened corneal area corresponding to the area of the leukoma on slit‐lamp photograph (D).
**Case 2:** A 5‐day‐old child was referred because of suspected left cataract. Anterior segments (AS) examination showed bilateral paracentral corneal opacities (Fig. 2D). A tentative diagnosis of Peter's anomaly was made. At the last assessment, biomicroscopy showed structural changes of iris and a round white structure located underneath the endothelium anteriorly to the limbus bilaterally. No cataract was seen. At this point, diagnosis was revised to Axenfeld's anomaly. Optical coherence tomography (OCT) showed a heterogeneous cornea (Fig. 2F) bilaterally with a smooth epithelial contour and uneven endothelium corresponding to the severe corneal opacities on slit‐lamp examination (Fig. 2D). The patient had a ‘band’ around the entire iris bilaterally forming a ‘ring’ located 1–2 mm from the trabecular meshwork.

**Case 1:** A 5‐year‐old patient was seen because of conjunctivitis. An irregular circular white structure located underneath of the endothelium anteriorly to the limbus was found incidentally (Fig. 2). Diagnosis of Axenfeld's anomaly was made. Optical coherence tomography (OCT) angle scans demonstrated a prominent anteriorly displaced Schwalbe line. The patient had several small (less than 1 mm) zones of the iridocorneal adhesions originating from the angular part of the iris and fixed behind the displaced Schwalbe line (Fig. 2E, right) in both eyes.

The AS OCT findings allowed diagnosis of iridocorneal endothelial syndrome (ICE) because of endothelium irregularity and the absence of iridocorneal adhesions and embryotoxon.

**Case 3:** A 2‐month‐old baby was referred due to a left corneal opacity. Investigation under GA showed iridocorneal and corneolenticular adhesions associated with corneal/lens opacity.

**Figure 3:**
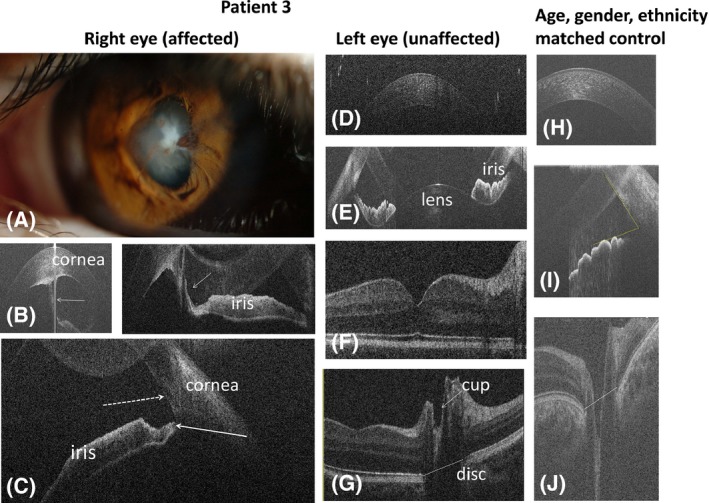
Anterior segment photography (A) and spectral domain optical coherence tomography (B–J) of case 3 with right Peter's anomaly (case 3; A–C = affected eye, D–G = unaffected eye) and a healthy control (H–J). Slit‐lamp photography (A) shows central corneal opacity with iridocorneal adhesion. The white arrows on figure B show iridocorneal adhesion on anterior OCT. On figure C, the dotted arrow indicates the scleral spur and the white arrow shows thinning of the iris root. Due to lens opacity in the right (affected) eye, posterior OCT was not possible. The horizontal lines on figures G and J connecting the edges of retinal pigment epithelium show the disc diameter. Optical coherence tomography (OCT) of the left (unaffected) eye of the patient with Peter's anomaly showed normal anterior segment structures (D, E) and macula (F) with small optic nerve cup (G) as compared to the control subject (J).

Optical coherence tomography (OCT) at the age of 6 years showed an irregular corneal structure with pericentral corneal thickening on the affected side (Fig. 3). An iridocorneal adhesion began at the thickest part of corneal endothelium and entered into the pupillary part of the iris. Typical contraction folds of the iris were missing (Fig. 3B). The anterior angle in the affected right eye (RE) was open and the iris root appeared thinner (Fig. 3C) than in the left eye (LE) (Fig. 3E) and healthy control (Fig. 3I). On the unaffected side, in the patient with transparent cornea, cup diameter and cup depth were smaller as compared to the control.

**Case 4:** A 6‐month‐old child presented due to a family history of *PAX6* gene mutation. Examination showed the presence of aniridia and small central lens opacities bilaterally.

**Figure 4:**
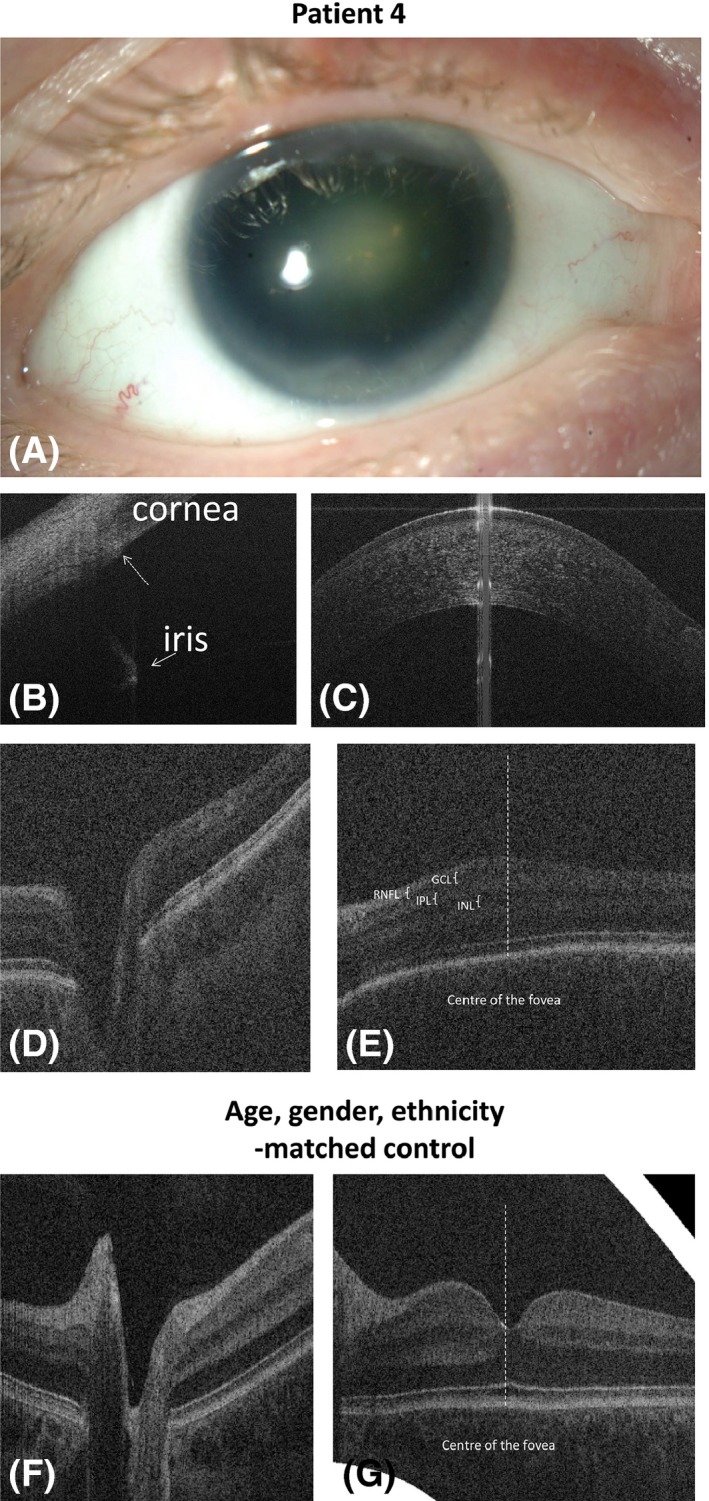
Anterior segment photography (A) and spectral domain optical coherence tomography (B‐G) of the right eye of case 4 with PAX6 mutation (case 4, A‐E) and a healthy control (F, G). Anterior segment photography (A) shows absence of the iris with lens. White arrow on the figure B: hypoplastic iris stump; dotted arrow: scleral spur; figure C shows normal cornea; Optic nerve tomograms of the patient with PAX6 mutation (D) and the healthy control subject (F); figure E shows the continuation of retinal nerve fibre layer (RNFL), ganglion cell layer (GCL), inner plexiform layer (IPL) and inner nuclear layer (INL) in the central foveal area (foveal hypoplasia) as compared to the normal fovea (G).

On OCT (Fig. 4), aniridia was visualized by a missing central part of the iris. A small flat hypoplastic iris stump was detected 360° around the anterior chamber. The patient had clearly defined scleral spurs (Fig. 4B, dotted arrow). Posterior examination showed severe foveal hypoplasia with continuation of retinal nerve fibre layer (RNFL), ganglion cell, inner nuclear and inner plexiform layers in the fovea bilaterally (Fig. 4E).

**Case 5:** A 3‐month‐old patient presented because of irregular pupil. Clinical examination showed left iris and choroidal coloboma. Optical coherence tomography (OCT) showed hypoplastic irides with a remaining iris stump at the area of the coloboma.

**Figure 5:**
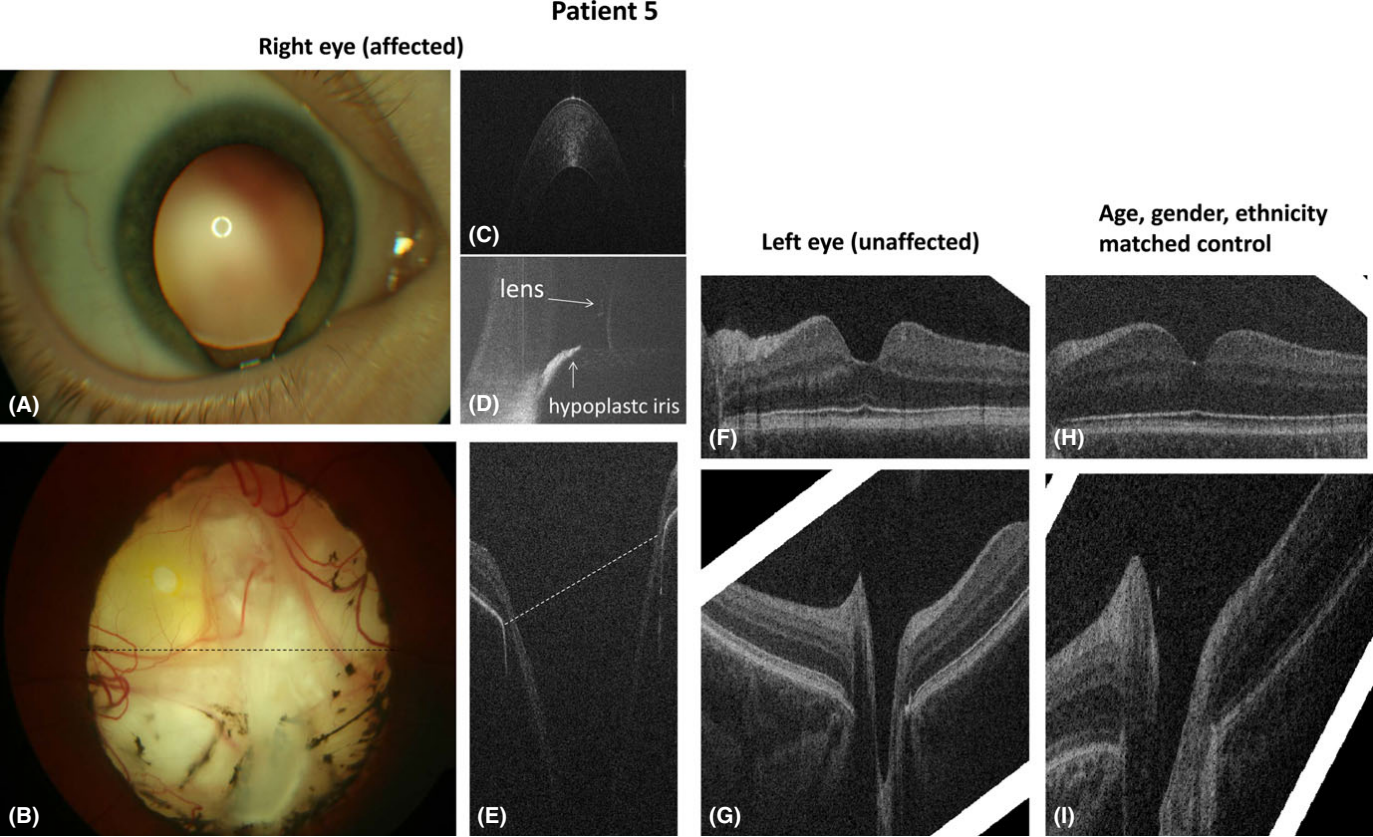
Anterior (A) and posterior (B) segment photography and OCT (C–J) of case 5 with right iris and optic nerve coloboma (case 5, A–G) and of the healthy control (H, I). Anterior segment photography (A) shows iris coloboma at 6 o'clock with visible zonula fibres of the lens; fundus photograph (B) demonstrates right optic nerve coloboma without clearly defined optic disc margins, and the fovea was not identified. Dotted lines in figures B and E display the corresponding area of the coloboma on fundus photography and OCT. On OCT, the optic nerve has a large excavation; the fovea was not seen. Figure C shows a normal cornea in the affected eye; figure D shows the anterior tomogram in the area of the coloboma with a flat hypoplastic iris stump. The fovea in the unaffected side (F) had a wider and larger foveal pit as compared to the healthy control (H). Tomograms of the optic nerve in the unaffected side (G) and healthy control (I) appeared similar.

A horizontal tomogram through the centre of the coloboma (Fig. 5E) showed the optic nerve (ON) with a significantly larger distance between the edges of RPE. Optic nerve (ON) cup diameter and depth in the affected eye were larger and nasal and temporal RNFL were thinner than in the fellow/control eye. Foveal pit was also larger in the unaffected eye (Fig. 5F).

**Figure 6:**
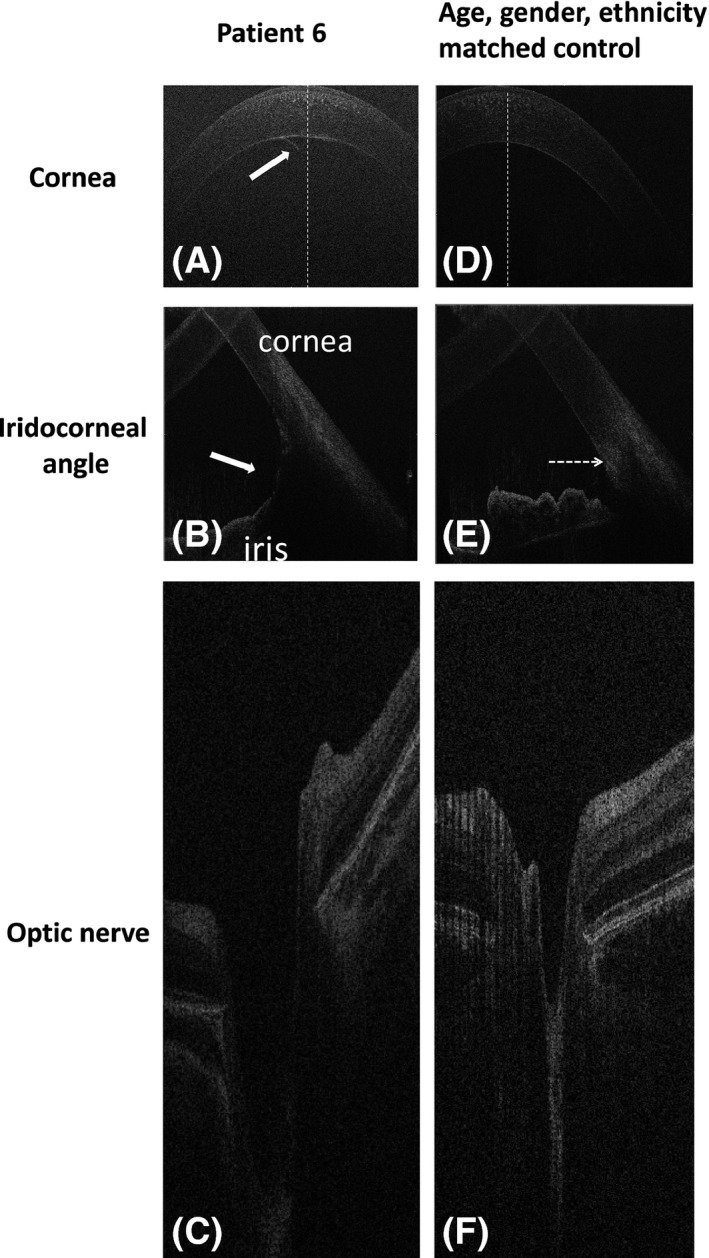
Spectral domain optical coherence tomography of case 6 with primary childhood glaucoma in the right eye (A–C) and the healthy control (D–F). The white arrow on figure A shows additional tissue on the endothelium in the centre of the cornea; arrow on figure B indicates abnormal iris insertion (iris root in front of the Schlemm canal) when compared to control; the horizontal tomogram of the optic nerve in the patient with glaucoma showed a larger horizontal diameter and depth of the cup as compared to the healthy control (F).

**Case 6:** A 4‐month‐old patient was referred because of a larger RE. Eye examination under GA revealed asymmetrical horizontal corneal diameters with right corneal haze and Haab's striae. Gonioscopy showed abnormal iris insertion with iris root seen in front of the trabecula meshwork. Optic nerve (ON) assessment showed an enlarged cup in the RE with a cup/disc ratio of 0.6. Diagnosis of primary congenital glaucoma (PCG) was established. Hand‐held optical coherence tomography (HH‐OCT) at the age of 4 years showed the presence of an additional tissue on the endothelium in the centre of the right cornea (arrow on the Fig. 6A) that was not seen on clinical assessment. Optical coherence tomography (OCT) also confirmed abnormal iris insertion (Fig. 6B). The horizontal cup diameter and maximal cup depth were larger in the glaucoma eye. Retinal nerve fibre layer (RNFL) thickness was approximately 15 *μ*m thinner in the eye with glaucoma.

Anterior OCT assessment enabled the pathology of the cornea to be determined in 4 of 6 patients that was not found clinically. In case 2, OCT findings dictated the necessity of long follow‐up of this patient as ICE is slowly progressive condition with extension of the endothelial membrane over the angle and high risk of subsequent glaucoma (Chandran et al. 2015).

Lai et al. (2013) described good visualization of the structures of the angle with swept‐source OCT and showed that it is possible to use OCT for risk assessment of adult angle‐closure glaucoma (Lai et al. 2013; Radhakrishnan & Yarovoy 2014; Sharma et al. 2014). We also found the OCT to be a useful technique in the assessment of the iridocorneal structure in case 6 with PCG, who had a large iridocorneal angle with insertion of the iris root in front of the Schlemm canal on OCT. To our knowledge, there are no data describing angle structure using OCT in patients with PCG. In PCG, patients often have some corneal opacification and Haab's striae (Patil et al. 2015). Abnormal iris insertion with the iris root starting in front of the Schlemm canal was clearly visualized on OCT. This indicates that OCT can be helpful in selecting the best surgical techniques using goniotomy and trabeculotomy if angle structure is preserved and drainage devices in severe angle dysgenesis.

Optical coherence tomography (OCT) findings in cases 4 and 5 indicate that subclinical maldevelopment can be present in retinal structures in clinically unaffected eyes. Important clinically relevant information helping diagnosis and management can be obtained non‐invasively without GA or sedation with anterior and posterior OCT to assist diagnosis and management of anterior segment dysgenesis (ASDs) especially PCG.
